# Predicting Biochemical Recurrence After Robot-Assisted Prostatectomy with Interpretable Machine Learning Model

**DOI:** 10.3390/jcm14197079

**Published:** 2025-10-07

**Authors:** Tianwei Zhang, Hisamitsu Ide, Jun Lu, Yan Lu, Toshiyuki China, Masayoshi Nagata, Tsuyoshi Hachiya, Shigeo Horie

**Affiliations:** 1 Department of Urology, Graduate School of Medicine, Juntendo University, Tokyo 113-8421, Japan; t.zhang.rf@juntendo.ac.jp (T.Z.); j.lu.yl@juntendo.ac.jp (J.L.); lyan@juntendo.ac.jp (Y.L.); tchina@juntendo.ac.jp (T.C.); m-nagata@juntendo.ac.jp (M.N.); t.hachiya.wa@juntendo.ac.jp (T.H.); shorie@juntendo.ac.jp (S.H.); 2 Department of Innovative Longevity, Graduate School of Medicine, Juntendo University, Tokyo 113-8421, Japan; 3 Data Science and Informatics for Genetic Disorders, Graduate School of Medicine, Juntendo University, Tokyo 113-8421, Japan

**Keywords:** machine learning, robot-assisted radical prostatectomy, biochemical recurrence, SHAP value

## Abstract

**Background:** This study aimed to develop and evaluate machine learning (ML) models to predict biochemical recurrence (BCR) after robot-assisted radical prostatectomy (RARP). **Methods:** We retrospectively analyzed clinical data from 1125 patients who underwent RARP between July 2013 and December 2023. The dataset was divided into a training set (70%) and a testing set (30%) using a stratified sampling strategy. Five ML models were developed using the training set. Model performance was evaluated on the testing set using the area under the receiver operating characteristic curve (AUC), accuracy, sensitivity, specificity, and F1 scores. Additionally, model interpretability was assessed using SHapley Additive exPlanations (SHAP) values to determine the contribution of individual features. **Results:** Among the five ML models, the LightGBM model achieved the best prediction ability with an AUC of 0.881 (95%CI: 0.840–0.922) in the testing set. For model interpretability, SHAP values explained the contribution of individual features to the model, revealing that pathological T stage (pT), positive surgical margin (PSM), prostate-specific antigen (PSA) nadir, initial PSA, systematic prostate biopsy positive rate, seminal vesicle invasion (SVI), pathological International Society of Urological Pathology Grade Group (pGG), and perineural invasion (PI) were the key contributors to the predictive performance. **Conclusions:** We developed and validated ML models to predict BCR following RARP and identified that the LightGBM model with 8 variables achieved promising performance and demonstrated a high level of clinical applicability.

## 1. Introduction

As the second most frequently diagnosed cancer in men globally, prostate cancer (PCa) presents a significant health burden, with a particularly high incidence in Western countries [1,2]. For localized PCa, radical prostatectomy (RP) has long been considered the standard treatment, effectively controlling disease progression and reducing the risk of distant metastasis and local recurrence [3]. In recent years, robot-assisted radical prostatectomy (RARP) has gained widespread acceptance as the primary surgical approach, gradually replacing traditional open and laparoscopic surgeries due to its minimally invasive nature and superior postoperative outcomes, such as improved urinary continence and erectile function preservation [4,5]. However, despite advancements in surgical techniques, postoperative biochemical recurrence (BCR) remains a significant challenge in postoperative management. BCR is characterized by an elevation in postoperative prostate-specific antigen (PSA) levels, with recurrence rates ranging from 15% to 35% within 5 years to 10 years after surgery [6,7].

BCR serves as not only an indicator of disease progression but also a crucial predictor of distant metastasis. Notably, when BCR occurs within two years postoperatively, it is associated with poor prognosis [8]. Therefore, early identification of patients at high risk for BCR is of paramount clinical importance for optimizing individualized postoperative management strategies, delaying disease progression, and improving long-term survival outcomes. Several clinical and pathological factors associated with BCR have been identified, including initial prostate-specific antigen (iPSA), Gleason score (GS), pathological T stage (pT), positive surgical margin (PSM), seminal vesicle invasion (SVI), perineural invasion (PI) [9,10]. Since the introduction of RARP in Japan in 2006, the clinical application of this surgical technique has shown a significant growth trend and has quickly gained widespread acceptance among both Japanese medical professionals and patients. However, compared to Western countries, where multiple medical centers have reported oncological outcomes following RARP, clinical research in this field remains relatively scarce in Japan [11].

Machine learning (ML), a discipline within artificial intelligence, is distinguished by its capacity to build models that learn directly from data, thereby constantly refining their performance [12]. ML algorithms can accommodate diverse data configurations, assign appropriate weights, and quantify the predictive power of variable combinations, thereby facilitating comprehensive assessment of diagnostic and prognostic factors [13]. In recent years, ML has been extensively employed in diverse medical fields, including disease diagnosis, outcome prediction, therapeutic decision-making, and medical image analysis, demonstrating significant potential in advancing precision medicine and improving clinical decision support systems [14]. Furthermore, the integration of explainability frameworks, such as SHapley Additive exPlanations (SHAP), mitigates the “black-box” nature of ML models, allowing clinicians to interpret and apply ML-based predictions in clinical decision-making with greater confidence [15]. This study aims to make a significant contribution by developing and validating machine learning models on a large, single-center Japanese cohort. To identify the optimal modeling strategy, we systematically compare the performance of diverse algorithms. Furthermore, we leverage advanced SHAP analysis to provide transparent and individualized risk explanations. Our ultimate goal is to deliver a model that is not only high-performing and interpretable but also clinically applicable and specifically tailored to this patient population.

## 2. Materials and Methods

### 2.1. Study Design and Patients

We retrospectively collected the clinical and pathological data of patients who underwent RARP between July 2013 and December 2023 in the department of Urology at Juntendo Hospital, Tokyo. The study included patients with localized prostate cancer, diagnosed by biopsy pathology and magnetic resonance imaging (MRI). Eligible patients were in good general health, suitable for general anesthesia, and had no severe cardiovascular disease, major comorbidities, or detectable metastases. The postoperative surveillance protocol involved regular ultrasensitive PSA measurements according to the following schedule: at 1 and 3 months, every 3 months for the first two years, every 6 months during the third year, and then annually. BCR was defined according to diagnostic criteria as two successive PSA ≥ 0.2 ng/mL following RARP. Patients were excluded if they had received neoadjuvant hormone therapy prior to RARP, lacked follow-up data, or failed to achieve a PSA level < 0.2 ng/mL after RARP. A stratified random sampling approach was employed to split the dataset into a training cohort (70%) and a testing cohort (30%). The study protocol was approved by our institution’s ethics committee and adhered to the principles of the Declaration of Helsinki.

### 2.2. Data Collection

The target variable for our predictive models was the occurrence of BCR during the follow-up period. To predict this outcome, clinical and pathological data on the patients included: age, body mass index (BMI), initial prostate-specific antigen (iPSA), prostate volume, prostate-specific antigen density (PSAD), testosterone (TST), prostate-specific antigen nadir (PSA nadir), systematic prostate biopsy positive rate, clinical T stage (cT), pathological T stage (pT), positive surgical margin (PSM), seminal vesicle invasion (SVI), perineural invasion (PI), pathological International Society of Urological Pathology Grade Group (pGG), and the D’Amico risk classification.

### 2.3. Feature Selection

Our statistical approach began with evaluating intergroup differences in clinical characteristics using Mann–Whitney U and chi-square tests (significance: *p* < 0.05, two-tailed). To distill the most informative predictors from the candidate variables, we implemented a LASSO regression model on the training data. The choice of LASSO was motivated by its dual function of maintaining strong predictive performance while ensuring model parsimony and interpretability for clinical application. A necessary preprocessing step involved standardizing all continuous variables with a Z-score transformation to address the model’s sensitivity to variable scaling. The L1 regularization inherent in LASSO shrank the coefficients of non-contributory features to zero, thus performing automated feature selection and yielding a more robust predictor subset. This method also effectively mitigated multicollinearity (screened using Spearman correlation) by retaining a single variable from any set of highly inter-correlated features.

### 2.4. Model Building

The patient cohort was partitioned into a training dataset (70% of patients) and a testing dataset (30%). This division was stratified based on the primary outcome (BCR status) to maintain the original event rate in both subsets. We then developed and compared five distinct machine learning models: Logistic Regression (LR), K-Nearest Neighbors (KNN), Multilayer Perceptron (MLP), Support Vector Machine (SVM), and Light Gradient Boosting Machine (LightGBM). Model training was performed exclusively on the training set, while the testing set was reserved for evaluating discriminative performance. This performance was quantified by the area under the receiver operating characteristic curve (AUC) and its 95% confidence interval (CI). The algorithm that yielded the highest AUC was ultimately selected as the optimal model.

### 2.5. Model Interpretation

To address concerns regarding the black-box nature of ML algorithms, we employed SHAP values to enhance the interpretability of our models. SHAP, including KernelSHAP and TreeSHAP, provided a novel approach for explaining and visualizing the predicted outcomes of various ML models [15]. TreeSHAP was a fast and precise algorithm designed to efficiently compute SHAP values for individual decision trees and tree-based ensemble models [16]. Calibration performance was compared based on visual inspection of calibration curves. A calibration curve was plotted by comparing predicted probabilities to observed event rates in deciles of predicted risk. Decision curve analysis (DCA) was conducted to evaluate net clinical benefit at different threshold probabilities.

### 2.6. Statistical Analysis

A thorough analysis of missing data was conducted prior to model development. The incidence of missing values was low for any single variable (<5%), and no missing data were present for the primary outcome (biochemical recurrence status). To address this, we employed a mean/mode imputation strategy. Specifically, missing values for continuous variables were imputed using the mean, while those for categorical variables were imputed using the mode. For descriptive statistics, non-normally distributed continuous data were summarized as medians with interquartile ranges (IQR), while categorical data were reported as frequencies and percentages. To assess intergroup differences, we employed the Mann–Whitney U test and the chi-squared test, respectively. The threshold for statistical significance was set at a two-sided *p*-value of <0.05. The entire analytical pipeline was executed using Python (Python Software Foundation, version 3.7.12, Wilmington, DE, USA). We utilized the StratifiedShuffleSplit function from scikit-learn for data partitioning and leveraged its broader toolkit for machine learning model development. Core statistical procedures, including LASSO and correlation analysis, were performed using the scipy (version 1.7.3), numpy (version 1.21.6), and sklearn (version 1.0.2) packages.

## 3. Results

### 3.1. Patient Characteristics

**Figure 1:**
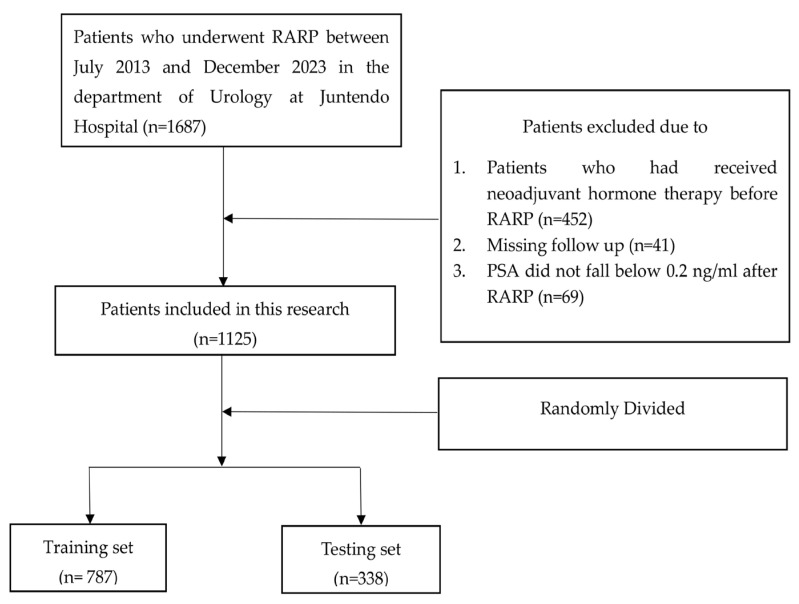
Flowchart illustrating the patient selection procedure.

Between July 2013 and December 2023, 1687 patients who underwent RARP at the Department of Urology, Juntendo Hospital, were initially eligible for inclusion. The median follow-up for the entire cohort was 48 months (IQR: 24–72 months). After the selection process, 1125 patients were enrolled: 178 patients with BCR and 947 patients without BCR. Using stratified random sampling, we split the cohort into the training set (70%) and the testing set (30%), yielding 663 patients without BCR and 124 with BCR in the training set and 284 without BCR and 54 with BCR in the testing set (Figure 1). The baseline characteristics were summarized in Table 1, revealing that iPSA, prostate volume, PSAD, PSA nadir, systematic prostate biopsy positive rate, D’Amico risk classification, pT, cT, PSM, PI, SVI, and pGG were significantly different between patients with and without BCR.

Table 2 provided a detailed comparison of the baseline characteristics between the training and testing cohorts. Our stratified sampling strategy successfully maintained a balanced distribution of the primary outcome, biochemical recurrence (BCR), across the sets (16.0% vs. 15.4%; *p* = 0.792). Moreover, the cohorts were found to be statistically comparable across all other baseline variables, thereby validating the creation of well-balanced populations for model training and evaluation.

### 3.2. Feature Selection

**Figure 2:**
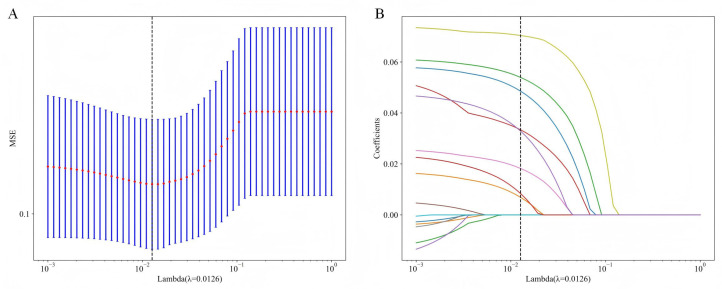
LASSO-based feature selection process and results. (**A**) Tenfold cross-validation is employed to tune the regularization parameter (λ), identifying an optimal value of 0.0126 that minimized the mean squared error (MSE). (**B**) This optimal λ results in the selection of eight features, as shown by their non-zero coefficients, which are subsequently used for model construction. LASSO, least absolute shrinkage and selection operator.

**Figure 3:**
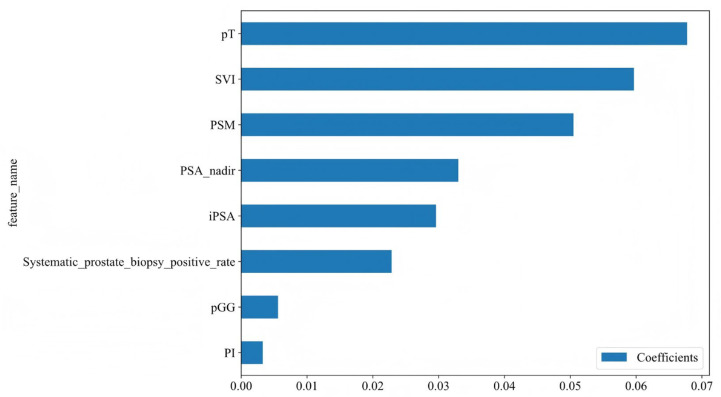
Feature coefficients for the eight variables identified through LASSO regression. The features are listed along the *Y*-axis. The *X*-axis represents the coefficient values for each feature. iPSA, initial prostate-specific antigen; PSA nadir, prostate-specific antigen nadir; pT, pathological T stage; SVI, seminal vesicle invasion; PSM, positive surgical margins; PI, perineural invasion; pGG, pathological International Society of Urological Pathology Grade Group.

Using Spearman correlation analysis and the LASSO algorithm, feature selection was performed to identify the most relevant variables. The optimal model was obtained when Lambda= 0.0126, achieving the best predictive performance. Through this process, the initial 15 variables were ultimately reduced to 8 key predictors for BCR following RARP (Figure 2A,B). As shown in Figure 3 and Table S1, our analysis identified eight potential predictors for BCR following RARP. These eight features were subsequently used to construct the predictive models.

### 3.3. Model Building and Performance Evaluation

**Figure 4:**
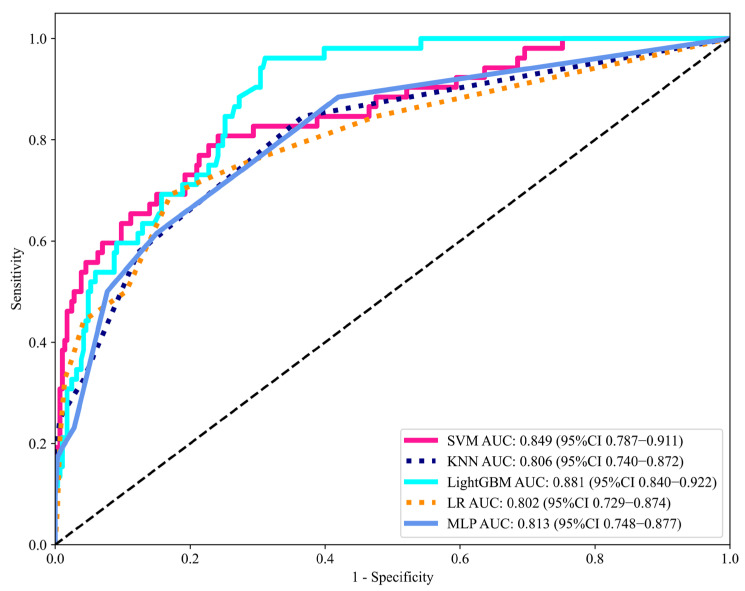
Discriminative performance of machine learning models in the testing set, evaluated by the area under the ROC curve (AUC). AUC, area under the curve; ROC, receiver operating characteristics; CI, confidence intervals; LR, Logistic Regression; KNN, K-Nearest Neighbor; MLP, Multilayer Perceptron; SVM, Support Vector Machine; LightGBM, Light Gradient Boosting Machine.

We developed five machine learning models using the eight selected features as input variables. The models were initially trained on the training set, and their predictive performance was subsequently validated on the testing set. Table 3 summarizes the AUC, accuracy, sensitivity, specificity, and F1 score for the testing set. The receiver operating characteristic (ROC) curves of each model in the testing set are presented in Figure 4. Among the algorithms, the LightGBM model demonstrated superior predictive performance, achieving an AUC of 0.881 (95% CI: 0.840–0.922) and sensitivity of 0.942 in the testing set. The calibration curve for the LightGBM model showed excellent agreement between predicted probabilities and observed outcomes in the testing set (Figure S1).

### 3.4. Interpretation of the Optimal Model with SHAP

**Figure 5:**
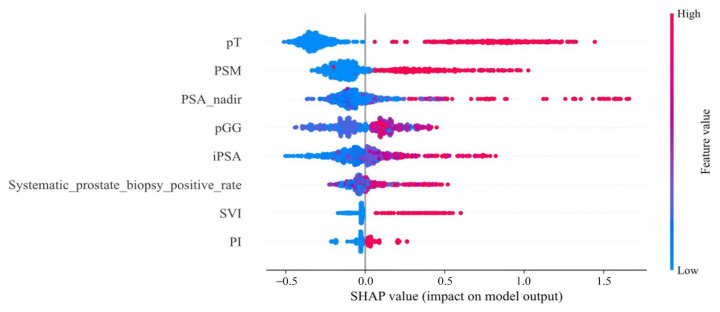
SHAP summary plot for the LightGBM model. This plot visualizes both the direction and magnitude of each feature’s effect on individual patient predictions from the testing set. Features are arranged along the *y*-axis. The *x*-axis quantifies a feature’s impact as a SHAP value, where positive values are associated with a higher predicted risk of BCR. The color of each data point represents the feature’s level for that patient, ranging from low (blue) to high (red). pT, pathological T stage; PSM, positive surgical margins; PSA nadir, prostate-specific antigen nadir; pGG, pathological International Society of Urological Pathology Grade Group; iPSA, initial prostate-specific antigen; SVI, seminal vesicle invasion; PI, perineural invasion.

**Figure 6:**
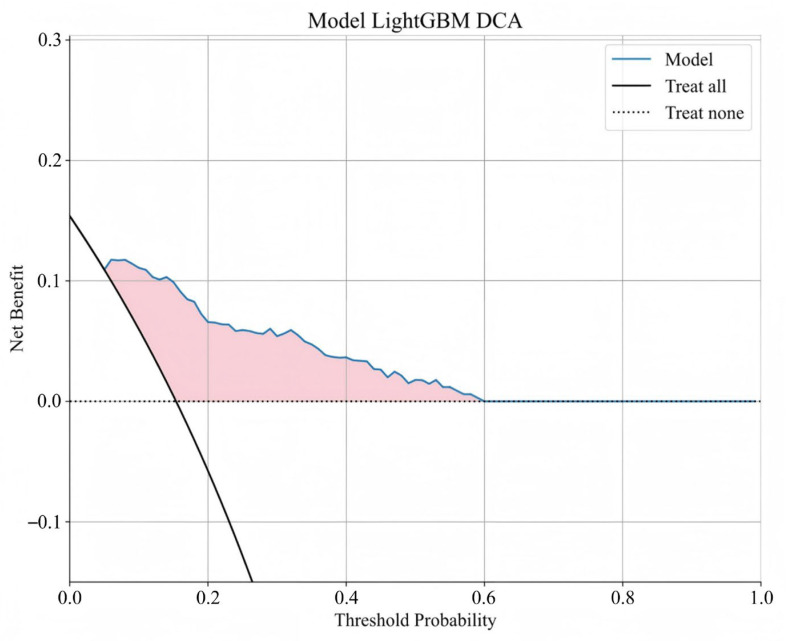
Decision curve analysis of LightGBM model. The *y*-axis signifies the net benefit, and the *x*-axis signifies the threshold probability. The solid black line indicates that all of the patients are treated, while the dashed black line indicates that none of the patients are treated.

The distribution of potential risk factors was visualized using SHAP summary plots with the LightGBM model (Figure 5). SHAP values were adopted to show the contribution of each predictor of BCR after RARP. Figure 5 highlighted eight key features incorporated in the LightGBM model, including pT, PSM, PSA nadir, pGG, iPSA, systematic prostate biopsy positive rate, SVI, and PI, all of which were positively associated with BCR following RARP. Furthermore, DCA was performed to evaluate the clinical utility of the LightGBM model. In the testing set, the model exhibited a positive net benefit over a wide spectrum of threshold probabilities, which underscored its potential value in supporting clinical decision-making (Figure 6).

## 4. Discussion

RARP has gained global acceptance in urological surgery due to its advanced technical advantages, including high-resolution three-dimensional visualization, magnified surgical field, enhanced precision and stability, and optimized operative conditions, which collectively contribute to improvements in functional recovery [17]. Compared to laparoscopic surgery, RARP has demonstrated superior outcomes in controlling urinary incontinence and preserving erectile function [18]. However, despite continuous advancements in RARP, postoperative BCR remains a major challenge in postoperative management, as it is closely associated with the prognosis and survival of prostate cancer patients. The early detection and prediction of BCR following RARP remain critical in optimizing postoperative management and improving the prognosis of prostate cancer patients [19]. Given that the median time from BCR to metastasis is approximately eight years, whereas the median survival time from metastatic PCa to mortality is only five years, the necessity of timely BCR prediction is paramount [8]. Our study represents a significant advancement in the prediction of biochemical recurrence following RARP for Japanese patients. For the first time, we have developed and validated machine learning models for the Japanese population, demonstrating that the LightGBM algorithm achieves superior predictive performance compared to other methods applied in this study. Moreover, our use of SHAP provides deep model interpretability, enhancing clinical trust and moving beyond simple ‘black-box’ prediction. This combination of a high-performing model and transparent interpretability provides significant clinical applicability and serves as a valuable resource for both clinicians and future research.

In our study, we initially employed LASSO for feature selection, followed by incorporating the selected features into the construction of machine learning models. LASSO, as a regularization method for feature selection and regression analysis, offers several advantages over conventional univariate and multivariate analysis in building machine learning prediction models: (1) LASSO automatically performs feature selection through L1 regularization, which shrinks the coefficients of less important features to zero, thereby identifying the most predictive features. (2) LASSO reduces multicollinearity effects through variable selection. (3) LASSO mitigates overfitting through regularization, thereby enhancing model performance on new datasets [20,21].

Accurate risk stratification is a cornerstone of modern prostate cancer management, as emphasized in the latest guidelines from both the European Association of Urology (EAU 2024) and the American Urological Association (AUA 2023) [22,23]. While these guidelines provide frameworks for treatment decisions based on broad risk categories, a significant need remains for more precise, individualized tools to predict BCR. The key predictors identified by our LASSO model, such as Gleason grade group, pathological T-stage, and surgical margin status, are recognized in the EAU 2024 guidelines as critical factors for risk assessment [22]. Furthermore, the development of predictive tools aligns with the AUA 2023 guidelines’ endorsement of risk calculators to facilitate shared decision-making [23]. By providing a clearer picture of their individual recurrence risk, our model can empower patients and clinicians in postoperative management discussions.

Machine learning models can accommodate non-linear relationships and integrate multimodal data, including clinical, pathological data, thereby providing a more comprehensive assessment of BCR risk. Previous studies have demonstrated the feasibility of ML for predicting postoperative BCR. For instance, Lu et al. reported that random forest model achieved an AUC of 0.846 in a cohort of 647 Chinese patients [24]. However, a critical gap has remained regarding its applicability to other populations. Our study addressed this gap by being the first to develop and externally validate ML-based predictors within a large, independent Japanese cohort of 1125 patients. This provides crucial, population-specific evidence and a tailored predictive tool for this demographic. Five ML-based predictive models were developed in our study: LR, KNN, MLP, SVM, and LightGBM. Among these models, the LightGBM model demonstrated the best predictive performance, achieving an AUC of 0.881 (95% CI: 0.840–0.922) in the testing set, making it an ideal choice for predicting BCR following RARP. The LightGBM model has demonstrated superior predictive performance through its integration of gradient boosting techniques, regularization methods, and optimized tree-structured architecture.

While our LightGBM model achieved the highest overall discriminative ability with an AUC of 0.881, we must acknowledge the important trade-off between its performance metrics. The model was optimized for a high sensitivity (0.942) at the expense of a more modest specificity (0.692) and accuracy (0.731). This performance profile indicates that while the model is highly effective at identifying patients who will experience BCR, it also generates a higher rate of false positives. This clinical trade-off is critical. Failing to identify a patient at high risk of recurrence (a false negative) could delay necessary adjuvant therapies and lead to poorer outcomes. Conversely, a false positive would primarily result in heightened surveillance (e.g., more frequent PSA monitoring). Therefore, our model should not be interpreted as a definitive diagnostic tool, but rather as a risk stratification instrument to identify patients who may benefit from closer follow-up. Clinical decisions regarding intervention must still integrate these predictions with comprehensive clinical judgment.

One of the primary challenges in adopting ML models in clinical practice is their perceived “black-box” nature. To overcome this challenge, we employed SHapley Additive ExPlanations (SHAP) to interpret and visualize the contributions of individual predictors in our model [25]. SHAP analysis revealed that the model’s predictions were primarily driven by pT, PSM, PSA nadir, pGG, initial PSA, systematic prostate biopsy positive rate, SVI, and PI, highlighting their critical roles in predicting BCR following RARP. This quantitative explanation helps build clinician trust and facilitates more meaningful dialogue with patients regarding their personalized risk profile. Therefore, the value of SHAP in our study is in making our high-performance model not just accurate, but also transparent and clinically intelligible.

Clinicians can input the patient-specific variables required by the model (such as PSA nadir, Gleason score, pathological stage, and surgical margin status). After submission, the LightGBM model calculates each patient’s risk of BCR. Importantly, SHAP is then used to explain this prediction by identifying the main factors driving the risk. For example, a patient with several favorable features (low Gleason score, negative margins) but one adverse factor (high PSA nadir) may be classified as moderate risk. SHAP clarifies that the elevated risk is almost entirely due to the high PSA nadir, despite other favorable features. This patient-specific explanation helps clinicians understand why the model makes its prediction, supports a tailored management strategy such as a more frequent PSA monitoring schedule, and builds trust by moving the model beyond a black-box.

Our findings are highly consistent with a large body of literature, indicating that initial PSA levels and postoperative PSA nadir are important predictors of BCR [26,27]. Initial PSA serves as critical biomarker for both preoperative risk stratification and postoperative monitoring. Elevated initial PSA levels have been linked to higher tumor burden and an increased risk of recurrence [28]. Extending beyond preoperative parameters, postoperative PSA nadir has been established as a critical prognostic indicator reflecting the completeness of surgery and the presence of residual tumor burden. Failure of the PSA nadir to reach an ideal level often indicates residual malignant prostate tissue or micro-metastases, thereby increasing the risk of postoperative BCR. Chung et al. revealed that a higher PSA nadir at 1 or 3 months after radical prostatectomy was associated with an increased risk of BCR [27]. The study by Liu’s group demonstrated that the PSA nadir was an effective predictor for BCR in the high-risk group following primary whole-gland prostate cryoablation [29]. For patients with elevated preoperative iPSA and a higher postoperative PSA nadir, our model’s risk stratification can help identify the need for more intensive postoperative monitoring, thereby optimizing postoperative patient care.

Similar to previous studies, we found that PSM, SVI and PI were pathological markers associated with BCR after RARP. PSM reflected the presence of residual cancer cells at the surgical margin, which often indicated incomplete tumor resection or residual microscopic invasive foci and was an important cause of persistently elevated postoperative PSA levels and early recurrence. Multiple studies consistently reported that PSM was an independent risk factor for BCR, and its presence significantly increased the risk of disease recurrence [26,30]. SVI, characterized by tumor extension beyond the prostatic capsule into the seminal vesicles, represented an advanced stage of local tumor spread and was strongly associated with worse pathological features and prognosis [31,32]. PI was a common indication of tumor metastasis that could be detected in multiple malignancies, including prostate cancer. During the development of PI, tumor cells interacted intimately with neural components in the tumor microenvironment, establishing a perineural niche that supported their survival and invasion. PI was frequently linked to adverse clinicopathological features and was associated with poor clinical outcomes in patients with prostate cancer [33,34]. Therefore, incorporating these key pathological factors into predictive models may improve the accuracy of postoperative recurrence risk assessment.

It has been reported that pT and pGG may be associated with BCR following RARP [35]. Advanced pathological stages, particularly pT3 or higher, were indicative of extraprostatic extension, greater tumor aggressiveness, and an elevated risk of recurrence [36]. Higher pGG scores were associated with more aggressive tumor behavior and an increased risk of recurrence. Ahove et al. previously identified pT and pGG as key prognostic factors for BCR following radical prostatectomy in patients with PCa [37]. Blas et al. also confirmed that pT and pGG were key contributors to BCR risk, highlighting the importance of tumor stage and histological aggressiveness in shaping postoperative outcomes [38].

Multiple studies have shown that a higher biopsy positive rate is closely associated with adverse pathological features, including higher Gleason scores, perineural invasion and metastases to regional lymph nodes [39]. The systematic prostate biopsy positive rate has recently emerged as a clinically meaningful prognostic biomarker. Unlike simply recording the number of positive cores, the positive rate corrects for sampling variability, thereby providing a more reliable estimate of total tumor burden. Moreover, it serves as an important predictor of increased BCR risk following definitive treatment [27]. Hence, biopsy positive rate should be incorporated into predictive models to improve their accuracy.

Despite its promising findings, this study has several limitations. First, a primary limitation of our study is its retrospective, single-center design, which inherently constrains the generalizability of our findings. Our findings were derived from a single institutional cohort and may not be directly generalizable to other populations due to inherent heterogeneity in patient demographics, disease biology, clinical management practices, and follow-up protocols. Consequently, our model should be regarded as exploratory. Rigorous external validation using multicenter, prospective data is an essential next step before this tool can be considered for widespread clinical implementation. Second, the model’s predictive scope is limited by the variables included. We did not incorporate known prognostic factors such as lymph node status, comorbidities, and hematological biomarkers. While this was partly due to data availability, their absence likely restricts the model’s overall predictive ceiling. Future iterations should aim to integrate a more comprehensive set of predictors. Third, we did not benchmark our model against established tools such as the CAPRA-S score or the MSKCC nomogram. These models were originally developed in North American populations, and applying them directly to our Japanese cohort without recalibration would not represent a methodologically sound comparison. Instead, the primary aim of our study was to develop and validate a high-performing model specifically tailored to our patient population. Fourth, while our models demonstrate robust performance for predicting recurrence within the first few years post-surgery, their long-term predictive validity warrants confirmation through extended follow-up in future studies. Finally, we only collected traditional pathological features of RARP and standard clinical data. One recently published study collected new pathological characteristics and radiomics features using a deep learning algorithm and constructed a deep learning model to predict BCR in prostate cancer. Thus, microscopic pathological variables and radiomic features could be integrated to construct predictive models using deep learning in the future.

## 5. Conclusions

In this exploratory study, we developed and validated interpretable ML models to predict BCR after RARP. LightGBM showed the best performance based on eight key clinical and pathological features. SHAP analysis enhanced model transparency, clarifying how each variable influenced individual risk. Our findings highlight the feasibility of using interpretable ML for early risk stratification. Further external validation across diverse populations is essential to establish the model’s generalizability and clinical utility in guiding postoperative management.

## Figures and Tables

**Table 1:** Baseline characteristics of the patients.

Feature Name	No BCR	BCR	*p* Value
Age (years)	66.97 ± 6.56	67.34 ± 7.00	0.496
BMI (kg/m^2^)	24.24 ± 3.24	24.19 ± 2.57	0.839
Prostate volume (ml)	32.07 (24.55, 35.0)	29 (21.33, 33.3)	0.005
iPSA (ng/mL)	6.5 (5, 8.89)	8.165 (6.11, 12.66)	<0.001
PSAD (ng/mL^2^)	0.28 (0.18, 0.29)	0.29 (0.23, 0.42)	<0.001
PSA nadir (ng/mL)	0.004 (0.002, 0.007)	0.005 (0.002, 0.013)	<0.001
TST (ng/mL)	4.6 (3.46, 5.91)	4.675 (3.68, 6.17)	0.422
Systematic prostate biopsy positive rate	0.21 (0.13, 0.33)	0.31 (0.19, 0.5)	<0.001
cT, *n* (%)			<0.001
1	171 (18.1%)	25 (14%)	
2	748 (79%)	131 (73.6%)	
3	28 (3%)	22 (12.4%)	
pT, *n* (%)			<0.001
1	2 (0.2%)	0 (0%)	
2	755 (79.7%)	55 (30.9%)	
3	190 (20.1%)	123 (69.1%)	
pGG, *n* (%)			0.046
1 (3 + 3 = 6)	149 (15.7%)	21 (11.8%)	
2 (3 + 4 = 7)	406 (42.9%)	66 (37.1%)	
3 (4 + 3 = 7)	231 (24.4%)	59 (33.1%)	
4 (5 + 3, 4 + 4, 3 + 5 = 8)	109 (11.5%)	26 (14.6%)	
5 (5 + 4, 4 + 5, 5 + 5 = 10)	52 (5.5%)	6 (3.4%)	
PSM, *n* (%)			<0.001
No	722 (76.2%)	85 (47.8%)	
Yes	225 (23.8%)	93 (52.2%)	
PI, *n* (%)			<0.001
No	551 (58.2%)	60 (33.7%)	
Yes	396 (41.8%)	118 (66.3%)	
SVI, *n* (%)			<0.001
No	920 (97.1%)	128 (71.9%)	
Yes	27 (2.9%)	50 (28.1%)	
D’Amico risk classification, *n* (%)			<0.001
Low	173 (18.3%)	21 (11.8%)	
Middle	604 (63.8%)	79 (44.4%)	
High	170 (18%)	78 (43.8%)	

BMI body mass index, iPSA initial prostate-specific antigen, TST, cT clinical T stage, pT pathological T stage, SVI seminal vesicle invasion, PSM positive surgical margins, PI perineural invasion, pGG pathological International Society of Urological Pathology Grade Group.

**Table 2:** Demographic comparison of the training and testing sets.

Feature Name	Training Set	Testing Set	*p* Value
Age (years)	68 (63, 72)	68 (63, 72)	0.932
BMI (kg/m^2^)	24.14 (22.17, 26.08)	23.72 (22.18, 25.53)	0.174
Prostate volume (ml)	32.07 (24, 34.55)	30.9 (23.4, 35)	0.191
iPSA (ng/mL)	6.73 (5.15, 9.41)	6.825 (5.12, 9.21)	0.893
PSAD (ng/mL^2^)	0.29 (0.18, 0.3)	0.285 (0.19, 0.33)	0.574
PSA nadir (ng/mL)	0.004 (0.002, 0.008)	0.004 (0.002, 0.007)	0.911
TST (ng/mL)	4.6 (3.49, 5.89)	4.68 (3.51, 6.15)	0.648
Systematic prostate biopsy positive rate	0.25 (0.13, 0.38)	0.25 (0.14, 0.4)	0.195
cT, *n* (%)			0.812
1	134 (17%)	62 (18.3%)	
2	619 (78.7%)	260 (76.9%)	
3	34 (4.3%)	16 (4.7%)	
pT, *n* (%)			0.584
1	2 (0.3%)	0 (0%)	
2	563 (71.5%)	247 (73.1%)	
3	222 (28.2%)	91 (26.9%)	
pGG, *n* (%)			0.406
1 (3 + 3 = 6)	119 (15.1%)	51 (15.1%)	
2 (3 + 4 = 7)	326 (41.4%)	146 (43.2%)	
3 (4 + 3 = 7)	204 (25.9%)	86 (25.4%)	
4 (5 + 3, 4 + 4, 3 + 5 = 8)	91 (11.6%)	44 (13%)	
5 (5 + 4, 4 + 5, 5 + 5 = 10)	47 (6%)	11 (3.3%)	
PSM, *n* (%)			0.617
No	568 (72.2%)	239 (70.7%)	
Yes	219 (27.8%)	99 (29.3%)	
PI, *n* (%)			0.479
No	422 (53.6%)	189 (55.9%)	
Yes	365 (46.4%)	149 (44.1%)	
SVI, *n* (%)			0.210
No	738 (93.8%)	310 (91.7%)	
Yes	49 (6.2%)	28 (8.3%)	
D’Amico risk classification, *n* (%)			0.082
Low	143 (18.2%)	51 (15.1%)	
Middle	461 (58.6%)	222 (65.7%)	
High	183 (23.3%)	65 (19.2%)	
BCR, *n* (%)			0.792
No	661 (84%)	286 (84.6%)	
Yes	126 (16%)	52 (15.4%)	

BMI body mass index, iPSA initial prostate-specific antigen, TST, cT clinical T stage, pT pathological T stage, SVI seminal vesicle invasion, PSM positive surgical margins, PI perineural invasion, pGG pathological International Society of Urological Pathology Grade Group.

**Table 3:** Comparison of predictive performance across machine learning models in the testing set.

Model	Accuracy	AUC	95% CI	Sensitivity	Specificity	F1
LR	0.808	0.802	0.729–0.874	0.692	0.829	0.526
KNN	0.666	0.806	0.740–0.872	0.846	0.633	0.438
MLP	0.814	0.813	0.748–0.877	0.615	0.85	0.504
SVM	0.763	0.849	0.787–0.911	0.788	0.759	0.515
LightGBM	0.731	0.881	0.840–0.922	0.942	0.692	0.524

AUC, area under the curve; 95% CI, 95% confidence intervals; LR, Logistic Regression; KNN, K-Nearest Neighbor; MLP, Multilayer Perceptron; SVM, Support Vector Machine; LightGBM, Light Gradient Boosting Machine.

## Data Availability

All data analyzed in this study are available upon reasonable request from S. Horie and the corresponding author, Hisamitsu Ide.

## Supplementary Materials

The following supporting information can be downloaded at: https://www.mdpi.com/article/10.3390/jcm14197079/s1, Table S1: Feature selection results and coefficients for each feature; Figure S1: The calibration curves of LightGBM model in test cohort.

## Abbreviations

BCR	Biochemical recurrence
RARP	Robot-assisted radical prostatectomy
ML	Machine learning
LR	Logistic regression
KNN	K-nearest neighbor
MLP	Multilayer perceptron
SVM	Support vector machine
LightGBM	Light gradient boosting machine
LASSO	Least absolute shrinkage and selection operator
ROC	receiver operating characteristic
AUC	Area under the curve
SHAP	Shapley additive explanations
cT	Clinical T stage
pT	Pathological T stage
PSM	Positive surgical margin
SVI	Seminal vesicle invasion
PI	Perineural invasion
pGG	Pathological International Society of Urological Pathology Grade Group
PSA	Prostate-specific antigen
iPSA	Initial prostate-specific antigen
TST	Testosterone
DCA	Decision curve analysis
