# Supplementary material for: Predicting Biochemical Recurrence After Robot-Assisted Prostatectomy with Interpretable Machine Learning Model
Source: J Clin Med. 2025 Oct 7;14(19):7079. doi: 10.3390/jcm14197079 (PMC12524934; doi:10.3390/jcm14197079)
Supplement: Supplementary file 1 [file jcm-14-07079-s001.zip › jcm-3874736-supplementary.pdf]

Table S1. Feature selection results and coefficients for each feature

Variable name	Coefficient
pT	0.067752
SVI	0.059676
PSM	0.05048
PSA nadir	0.03298
iPSA	0.0296
Systematic prostate biopsy positive rate	0.022862
pGG	0.005597
PI	0.003283

pT pathological T stage, SVI seminal vesicle invasion, PSM positive surgical margins, iPSA initial prostate-specific antigen, pGG pathological International Society of Urological Pathology Grade Group, PI perineural invasion.

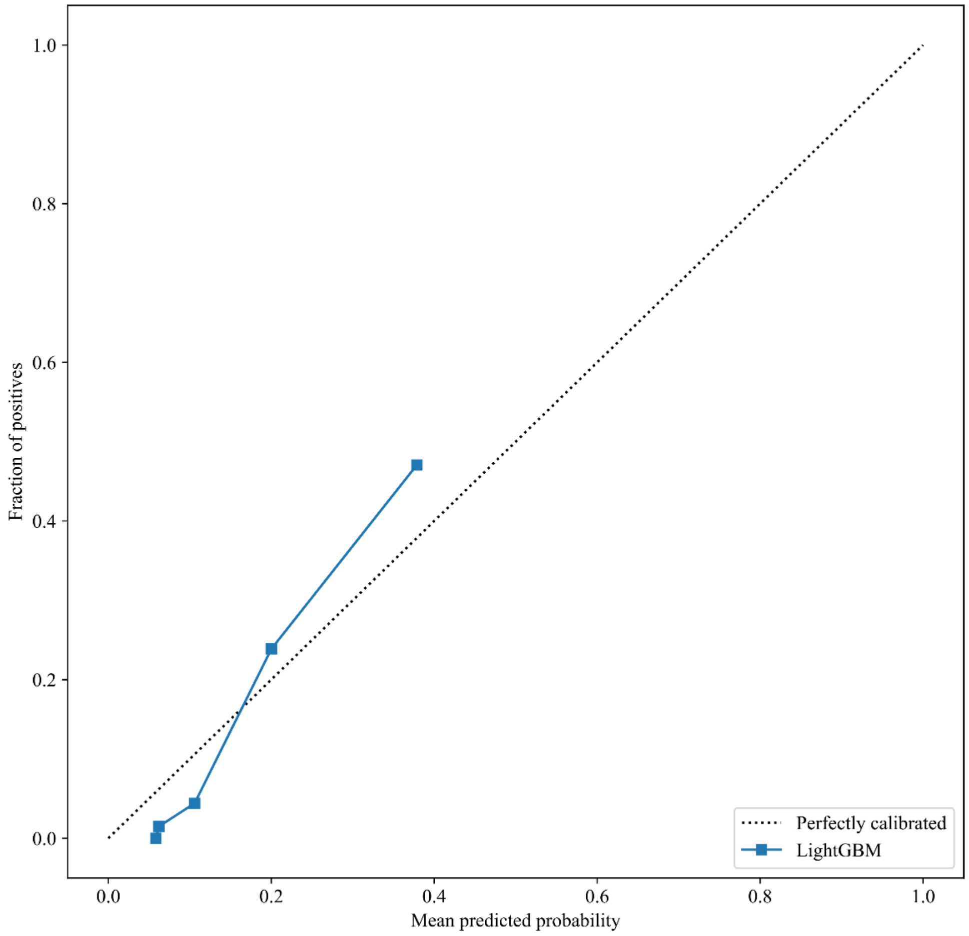

Figure S1. The calibration curves of LightGBM model in test cohort. LightGBM, light gradient boosting machine.
